# Epidemiology of severe pediatric adenovirus lower respiratory tract infections in Manitoba, Canada, 1991-2005

**DOI:** 10.1186/1471-2334-12-55

**Published:** 2012-03-13

**Authors:** Saleh Alharbi, Paul Van Caeseele, Raquel Consunji-Araneta, Taoufik Zoubeidi, Sergio Fanella, Abdul-Kader Souid, Ahmed R Alsuwaidi

**Affiliations:** 1 Umm Al-Qura University, Makkah, P.O. Box 6707, Saudi Arabia; 2 Department of Pediatrics and Child Health, University of Manitoba, Winnipeg, MB R3T 2N2, Canada; 3 Cadham Provincial Laboratory, Winnipeg, MB R3E 3J7, Canada; 4 Department of Statistics, United Arab Emirates University, Al Ain, P.O. Box 17666, United Arab Emirates; 5 Department of Pediatrics, United Arab Emirates University, Al Ain, P.O. Box 17666, United Arab Emirates

## Abstract

**Background:**

Most pediatric adenovirus respiratory infections are mild and indistinguishable from other viral causes. However, in a few children, the disease can be severe and result in substantial morbidity. We describe the epidemiologic, clinical, radiologic features and outcome of adenovirus lower respiratory tract infections (LRTI) in Aboriginal and Non-Aboriginal children in Manitoba, Canada during the years 1991 and 2005.

**Methods:**

This was a retrospective study of 193 children who presented to the department of pediatrics at Winnipeg Children's Hospital, Manitoba, Canada with LRTI and had a positive respiratory culture for adenovirus. Patients' demographics, clinical and radiologic features and outcomes were collected. Adenovirus serotype distributions and temporal associations were described. Approximate incidence comparisons (detection rates) of adenovirus LRTI among Aboriginal and Non-Aboriginal children were estimated with 95% confidence intervals.

**Results:**

Adenovirus infections occurred throughout the year with clusters in the fall and winter. Serotypes 1 to 3 were the predominant isolates (two thirds of the cases). The infection was more frequent among Canadian Aboriginals, as illustrated in 2004, where its incidence in children 0-4 years old was 5.6 fold higher in Aboriginals (13.51 vs. 2.39 per 10,000, *p *< 0.000). There were no significant differences in length of hospitalization and use of ventilator assistance between the two groups (*p *> 0.185 and *p *> 0.624, respectively) nor across serotypes (*p *> 0.10 and *p *> 0.05, respectively). The disease primarily affected infants (median age, 9.5 months). Most children presented with bronchiolitis or pneumonia, with multi-lobar consolidations on the chest x-ray. Chronic (residual) changes were documented in 16 patients, with eight patients showing bronchiectasis on the chest computerized tomography scan.

**Conclusions:**

Adenovirus infection is associated with significant respiratory morbidities, especially in young infants. The infection appears to be more frequent in Aboriginal children. These results justify a careful follow-up for children with adenovirus LRTI.

## Background

Adenoviruses play a significant role in pediatric infections, accounting for 2-5% of the overall respiratory illnesses and 4-10% of the pneumonias [1]. Fifty-six adenovirus serotypes are known so far and are classified into 7 species (species A to G) on the basis of serology, whole genome sequencing and phylogenomics [2-6].

The subspecies B1 includes serotypes 3, 7 and 21, which are responsible for most of the adenovirus lower respiratory tract infection (LRTI) epidemics [7]. The species C includes serotypes 1, 2, 5 and 6, and the species E includes serotype 4. These serotypes are also common causes of pediatric upper and lower respiratory infections [8-14].

In otherwise healthy children, most adenovirus respiratory infections are mild and indistinguishable from other viral causes. However, specific serotypes, such as serotypes 3, 7 and 14, have been implicated with fatal outcomes [15-17]. Severe disease has also been reported among newborns and patients with underlying medical conditions [18,19].

In a study of 41 patients with adenovirus bronchiolitis in Manitoba between 1974 and 1978, Wenman et al. depicted the natural history and emphasized the seriousness of adenovirus infections among young infants. Predilection of the disease in Canadian Aboriginal children (32 of 41 patients) was demonstrated, with an increased fatality rate in that group (5 of 41 patients). All Non-Aboriginal children survived. The authors suggested future studies to explore this issue [20].

This apparent susceptibility of Aboriginal children to adenoviruses needs to be confirmed and investigated further. Genetic, socio-economic and nutritional variables have been suggested [20,21]. Furthermore, it is unclear whether these findings will persist decades later. The primary aims of this study were to describe the epidemiologic, clinical, radiologic features and outcome of adenovirus LRTI in Aboriginal and Non-Aboriginal children in Manitoba, Canada during the years 1991-2005.

## Methods

### Patients and settings

The Aboriginal and Non-Aboriginal children live in different settings in Manitoba. In 2001, 34.7% of Manitoba's Aboriginal people lived within the municipal boundaries of the City of Winnipeg, while the remaining lived on-reserve scattered throughout the province or in other urban settings. Non-Aboriginal children live mainly in urban areas [28].

Despite their wide geographical distribution, both populations ultimately seek the same Children's Hospital.

### Study design

This retrospective study involved children 0 to 4 years of age who presented to the pediatric outpatient and emergency departments at Winnipeg Children's Hospital (Manitoba, Canada) between February 1991 and October 2005 with LRTI and positive respiratory culture for adenovirus.

The study sample was sourced from adenovirus culture positive specimens. Subsequently, a chart review (emergency department, outpatient and inpatient records) was done on those adenovirus positive culture cases and those with a diagnosis of LRTI were selected for analysis.

Recognition of pediatric LRTI was based on the physician diagnosis of pneumonia, bronchitis and bronchiolitis. (All medical codes in the Health Sciences Center Historical Abstracting System, WinRecs that contain the words "pneumonia", "bronchitis" or "bronchiolitis" were included.) Only the first adenovirus respiratory isolate, during a single clinical event, was included in the analysis. The studied cases did not include readmissions. Participants' demographic, clinical and radiologic findings were collected. Clinical outcome data were assessed according to days of hospitalization, use of mechanical ventilation, and mortality. Aboriginal ethnicity was identified by the demographic data. Approximate annual incidence (detection rate) of adenovirus LRTI among Aboriginal and Non-Aboriginal children was calculated from 1999 to 2005 as the ratio of the number of infected children 0-4 years old in the target year to the number of 0-4 year-old children in the corresponding ethnic group living in Manitoba in 2001 (i.e., 20,725 Aboriginals and 50,305 Non-Aboriginals) [28]. The numbers of 0 to 4 year-old children in 2001 were used as the base values in this calculation because 2001 is close to the median year of the study period (1999 - 2005) and the lack of data on the relevant population sizes in the other study years.

The study was approved by Bannatyne Campus Research Ethics Board at the University of Manitoba, Winnipeg, Canada (H2006:166).

### Specimens

Although testing for respiratory viruses was not a standard policy for all LRTI admissions during the study period, this was a usual practice. All nasopharyngeal aspirate specimens received at Cadham Provincial Laboratory (CPL), during the study period had received culture for adenovirus. Moreover, cultures for influenza A and B, parainfluenza 1, 2, 3, and 4, respiratory syncytial virus (RSV), respiratory enteroviruses and (on request) rhinoviruses were also done for all specimens. Rapid antigen detection for influenza A and RSV was conducted during their respective seasons. Influenza B rapid antigen testing was offered during any heavy B seasons.

Nasopharyngeal aspirate specimens were collected in virus transport media. During daytime, the samples were immediately transported at 4°C with a cold pack to CPL, which was adjacent to the Children's Hospital. At nights and weekends, the samples were stored at the microbiology laboratory of the Children's Hospital. Nevertheless, the procedures of sample collection and transportation were consistent throughout the study.

Specimens were processed as described [30]. Cultures were observed for 3 to 4 weeks for cytopathic effects or hemadsorption, and were further tested by indirect immunofluorescence staining. The various kits used throughout the study were conducted exactly according to manufacturer directions. Therefore, the impact of changes in laboratory techniques during this time is expected to be negligible.

Serotyping was performed by experienced staff. The common respiratory serotypes in children (types 1-7) were identified by neutralization assays with type-specific reference antisera [14,31]. Other serotypes were not tested.

### Statistical analysis

The data were analyzed using SPSS-PC software package, version 18.0 (IBM Corporation, NY, USA). Prevalence rate was calculated with 95% CI. Mann Whitney test was used to compare the median values of quantitative responses across groups and chi square was used for categorical variables. Simple logistic regression analysis was used to compare the use of mechanical ventilation across serotypes. Centered moving averages of the monthly serotype records were used to study seasonality of the incidence of serotypes. The F-test of linear trend in the simple linear regression was used to test the presence of a linear trend in the monthly records. A multiplicative time series model was assumed to identify the annual and monthly variations of the series of monthly incidences of all serotypes. For all these tests, statistical significance was defined for *p *< 0.05. The individual control chart (I chart) was used to detect whether the process of yearly incidences was stable.

## Results

### Epidemiology

One hundred ninety three children were identified with LRTI and positive respiratory culture for adenovirus (Table 1). The patients were predominantly infants (mean age ± SD = 11.1 ± 8.1 months). One hundred and twelve (58%) affected children were Canadian Aboriginals.

**Table 1:** Epidemiologic, clinical and virologic characteristics of 193 Manitoban children with Adenovirus LRTI

*Characteristics*	*n (%)*
Female	68 (35%)
Age (mo)	11.1 ± 8.1
Canadian Aboriginal	112 (58%)
History of prematurity (< 37 weeks gestation)	23 (12%)
Hospitalization	128 (66%)
Length of hospitalization (days)^#^	5 (1-189)
Clinical findings	
fever	128 (67%)
hypoxia	86 (46%)
rales	86 (45%)
wheezing	85 (45%)
conjunctivitis	13 (7%)
otitis media	19 (10%)
vomiting	39 (20%)
diarrhea	38 (20%)
Serotypes^¶^	
1	40 (22%)
2	53 (29%)
3	37 (20%)
4	6 (3%)
5	18 (10%)
6	5 (3%)
7	24 (13%)
Associated isolates	
RSV	11 (6%)
Parainfluenza	6 (3%)
Influenza A	1 (< 1%)
CMV	1 (< 1%)
*K. pneumoniae*	2 (< 1%)
*B. pertussis*	1 (< 1%)
Managements	
antibiotics	139 (72%)
salbutamol	127 (66%)
oxygen	88 (46%)
steroids	60 (31%)
mechanical ventilation	21 (11%)
Bronchiectasis	8 (4%)

Values are mean ± SD; otherwise, number (percent) of patients.

# Median is reported with (range).

¶ Ten isolates were un-typed.

The incidence of adenovirus infection in Aboriginals appeared to be higher than in Non-Aboriginals. The approximate incidence rate of adenovirus infection in children of 0 to 4 years per 10,000 per year, (see Methods) between 1999 and 2005 ranged from 0.097 to 1.351 for Aboriginals and 0.020 to 0.358 for Non-Aboriginals. For example, in 2004, the incidence among Aboriginals was about 5.6 fold higher than in Non-Aboriginals (1.351 vs. 0.239, *p *< 0.00).

**Figure 1:**
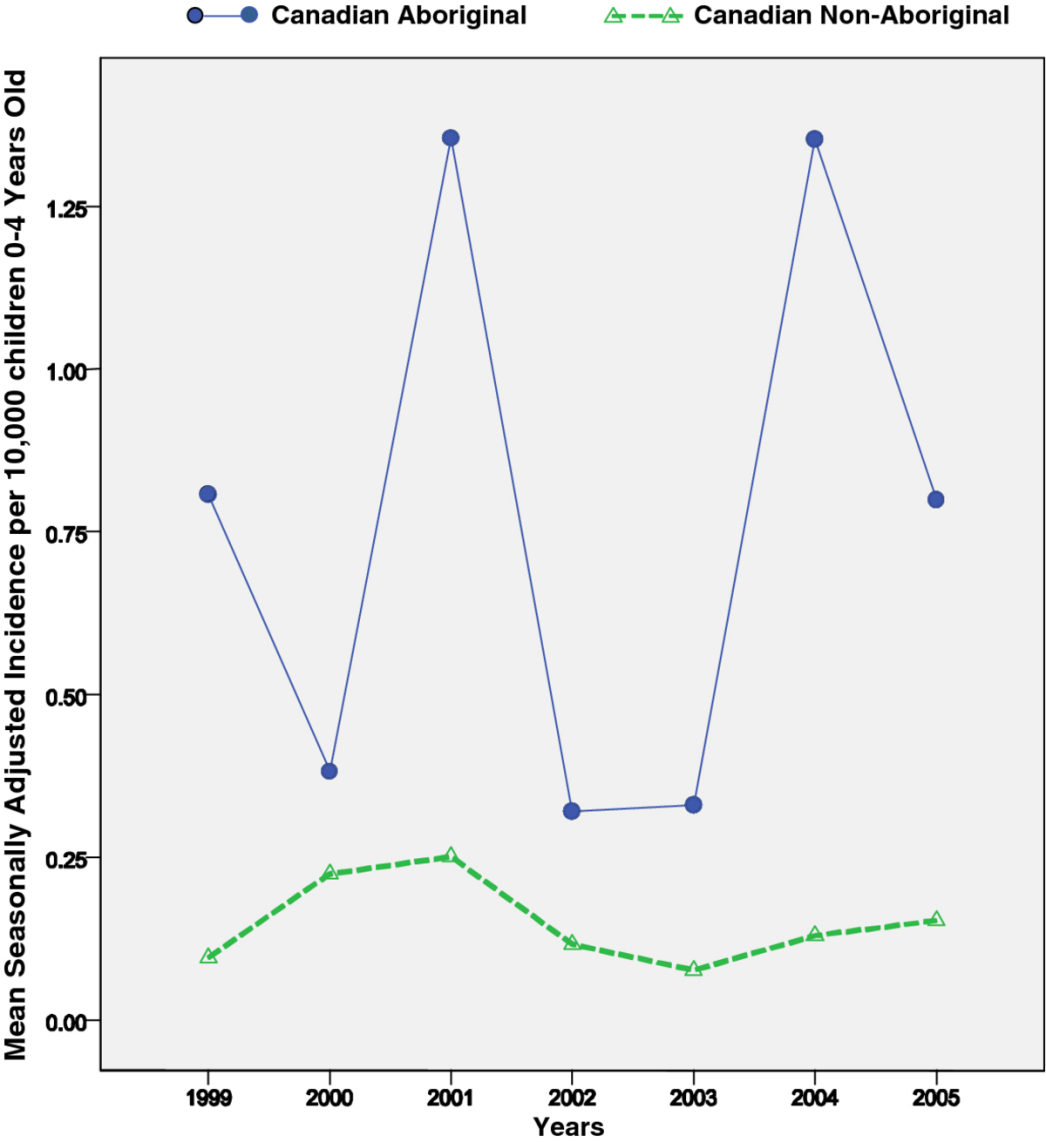
**Annual means of seasonally-adjusted series of monthly records of incidence of all serotypes among 0 to 4 year-old Aboriginal and Non-Aboriginal children**. The annual average of seasonally-adjusted incidence, that is, the incidence in each month was divided by the season's factor (months with high incidence had a factor > 1, while months with low incidence had a factor < 1) then the average for the 12 months of each year was computed.

Figure 1 shows the yearly variation in the incidence of adenovirus infection in Aboriginal and Non-Aboriginal children. It displays the average incidence after removing the season's effect (here, season = month). Higher incidences were noted in 2001 and 2004.

The series of monthly records of the incidence of serotypes appeared to be stationary, i.e., the incidence was relatively stable over the studied years (test of linear trend had *p *= 0.751 for Aboriginals and *p *= 0.382 for Non-Aboriginals).

Of note, from 1999 to 2005, there were 177 adenovirus isolates, representing 3.0% of total LRTI and 15.1% of confirmed viral LRTI.

### Virology

**Figure 2:**
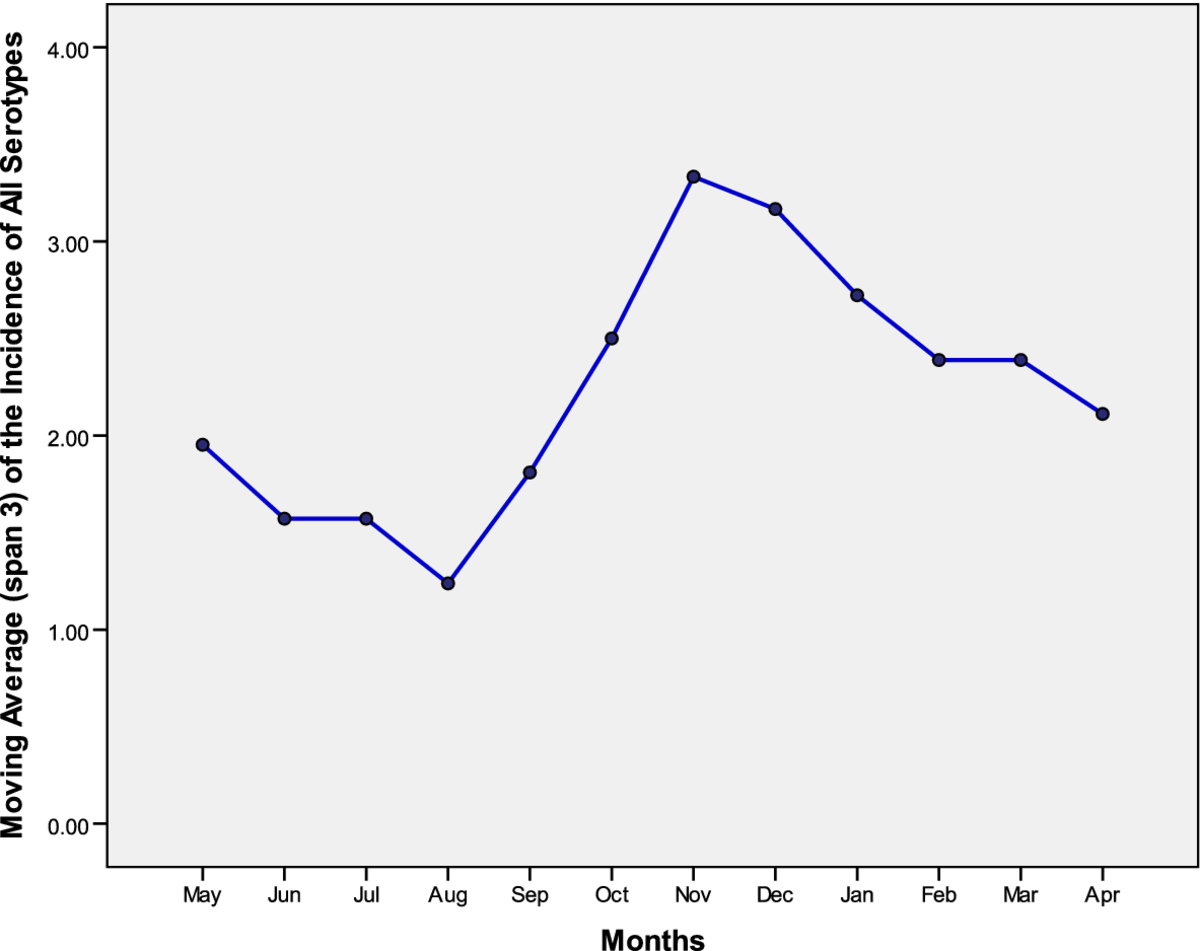
**The monthly averages of a centered-moving average (of span 3) of monthly records of all serotypes among 0 to 4 year-old children between 1999 and 2005**.

**Figure 3:**
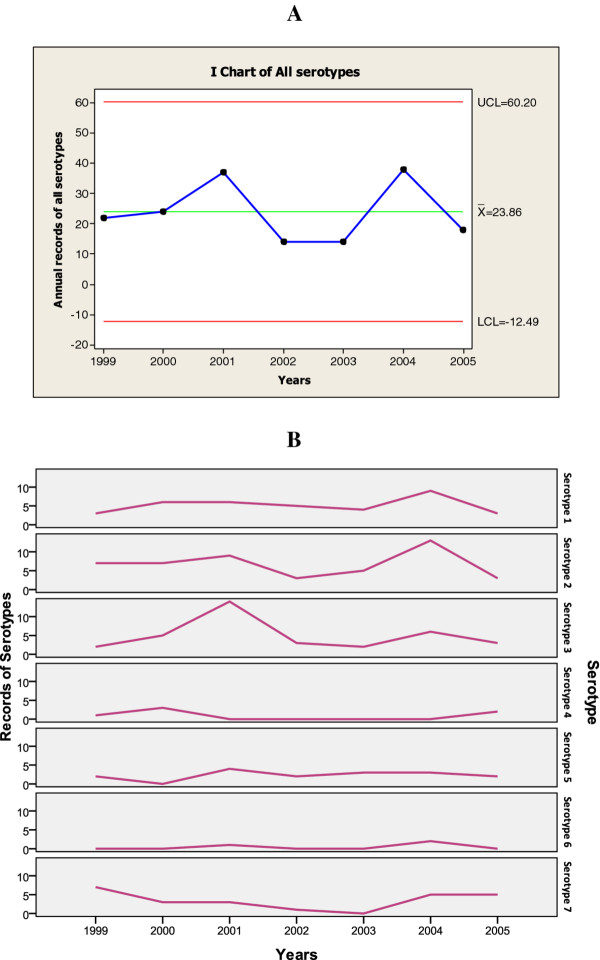
**Annual records (1999 to 2005) of all serotypes (Panel A) and of each serotype (Panel B) for 0 to 4 year-old children**.

During the study period, seven adenovirus serotypes were isolated from the nasopharyngeal aspirates (Table 1). Serotypes 1-3 were the most common, accounting for about two-thirds of the isolates. Ten isolates were un-typed. Although adenovirus infections occurred year-round, infections peaked in the fall and winter (Figure 2), especially serotypes 1-3, 5 and 7 (data not shown). Two outbreaks (notable rises in the incidence of infections) occurred (Figure 3A-B), one in 2001 (predominantly serotype 3) and one in 2004 (predominantly serotypes 2 and 1).

### Clinical data at presentation

Two-thirds of the children were hospitalized (median = 5 days, range = 1.0 to 189 days). Most of them had pneumonia (46%) or bronchiolitis (38%). Wheezing and hypoxia were documented in about half of the patients. Extra-pulmonary manifestations included vomiting (20%), diarrhea (20%) and conjunctivitis (7%).

Many patients received antibiotics (72%), bronchodilators (66%), oxygen (46%) and steroids (31%). Mechanical ventilation was used in 11%.

Two-thirds (141 of 193) of the patients had lobar consolidation on the chest x-ray. All lobes were equally affected and the involvement was multi-lobar in 56 (28%) and bilateral in 33 (17%) patients. Residual changes on follow-up evaluations were present in 16 patients, including bronchiectasis in 8 children.

Co-infections with other respiratory viruses were found in 19 (10%) patients (11 RSV, 6 parainfluenza, 1 influenza A and 1 CMV). The co-infection did not adversely affect the length of hospitalization (*p *= 0.096) or the incidence of bronchiectasis (*p = *0.797).

### Outcome

All patients were discharged home and there were no deaths. There were no significant differences between Aboriginal and Non-Aboriginal children in the length of hospitalization (*p *= 0.185) or the use of mechanical ventilation (of 21 patients who had ventilation, 11 were Aboriginal and 10 were Non-Aboriginal, *p = *0.624). Moreover, the length of hospitalization and the use of ventilation did not differ across serotypes (*p *= 0.612 and *p *= 0.072, respectively).

Only 27 (14%) of the 193 children had documented long-term outpatient follow-up and 11 (6%) had bronchoscopy. All children had chest x-ray and 10 had chest computerized tomography (CT) scan.

Bronchiectasis (single or multiple lobes) was diagnosed on the chest CT scan in eight patients (4%); three had serotype 1, two had serotype 3, one had each of the serotypes 2, 7 and 5 (Table 2). There was no statistically significant association between bronchiectasis and serotypes (*p *= 0.822). Bronchiectasis distributed equally among Canadian Aboriginal and Non-Aboriginal children (*p *= 0.638). The prevalence of bronchiectasis among both ethnic groups was estimated, with 95% confidence, between 0.02 and 0.08.

**Table 2:** Characteristics of the eight patients with bronchiectasis

Age (mo)	Gender	Ethnicity	Adenovirus Serotype	Admission	Location	Days of Hospitalization	House-hold Smoking	Management	Associated Diagnoses
11	male	Canadian Aboriginal	3	July 2001	Rt middle lobe	112	Yes	ventilation, oxygen, salbutamol, steroids, antibiotics	BOOP
5	female	Canadian Non-Aboriginal	2	Nov 2000	Lt lower lobe	12	No	oxygen, salbutamol, steroids, antibiotics	Asthma
11	male	Canadian Aboriginal	3	Dec 2002	Rt middle and lower lobes	8	No	oxygen, salbutamol, steroids, antibiotics	Asthma
16	male	Canadian Aboriginal	7	June 1999	Lt lower lobe	7	No	oxygen, salbutamol, steroids, antibiotics	No follow up
20	male	Canadian Non- Aboriginal	1	Dec 2000	Rt and Lt lower lobes	3	No	salbutamol, antibiotics	None
3	female	Canadian Non-Aboriginal	5	March 2004	Rt and Lt upper lobes Lt lower lobe	189	Yes	ventilation, oxygen, salbutamol, steroids, antibiotics	Asthma
16	male	Canadian Non-Aboriginal	1	Nov 2000	Rt upper and lower lobes	8	No	oxygen, salbutamol, steroids, antibiotics	None
8	male	Canadian Aboriginal	1	April 1999	Rt upper and middle lobes	25	No	ventilation, oxygen, salbutamol, steroids	BOOP

*BOOP *bronchiolitis obliterans with organizing pneumonia.

## Discussion

Adenovirus LRTI occurred endemically throughout the year, with clusters in the fall and winter. A seasonal peak in November-December was clearly noticeable (Figure 2). This result is consistent with a recent report from Western Australian Aboriginal and Non-Aboriginal children, showing a clear seasonality for adenovirus identifications [22]. Thus, adenoviruses should be considered all year long and surveillance programs should be in place to monitor peaks in infection rates.

In the studied population, serotypes 1-3 were the most commonly identified adenovirus serotypes (Table 1). This finding is consistent with reports from other geographic regions, including Korea, Texas and Toronto. [23-25]. However, 10 isolates were un-typed, possibly due to failure of the neutralization assay. Un-typed isolates could be non 1-7 serotypes, such as 14 or 21, which potentially circulate around that time period. Molecular methods for typing adenoviruses, such as restriction fragment length polymorphism (RFLP), PCR-based assays, microarray-based methods and phylogenetic analysis have now been established and may overcome the practical issues with the traditional neutralization assay. They are becoming more important for epidemiological surveillance, outbreak investigation, and detection of new strains as well as understanding the pathogenesis of adenovirus infection [26,27].

The infection rate was strikingly high among Canadian Aboriginals, considering their proportion in the studied population. While the total population of Manitoba has been relatively stable over the last 20 years, the Aboriginal population has been increasing due to a higher birth rate, combined with an Aboriginal mortality rate that is much lower than prior to 1981. The Aboriginals constituted 8.7% of the Manitoba population in 1986, 10.6% in 1991, 11.7% in 1996, 13.6% in 2001, and 15.5% in 2006. The percentage of Aboriginal children in 2001 was 25% of those 0-4 years of age and 28% in 2006 of those 0-4 years of age [28]. Earlier Manitoban and New Zealand experiences showed an increased occurrence of adenoviruses in Aboriginal children [20,21]. Our data support this finding and confirm its persistence since at least 1969. However, the outcome appears to be similar between the two groups.

Seventeen (15.2%) of 112 Aboriginal patients had house-hold smoking; while 10 (12.3%) of 81 Non-Aboriginal patients had house-hold smoking (*p *= 0.576). As previously suggested, factors that could contribute to a high predilection of adenovirus infection in Aboriginal children include socioeconomic, crowding and nutritional conditions [20,21]. Further studies are much needed to explore these risk variables.

The high prevalence (73%) of consolidation on chest x-ray may suggest bacterial co-infection. The possibility of viral/bacterial co-infection as a cause of the x-ray findings is thus realistic, especially since 72% of the patients received antibiotics (Table 1). Furthermore, the x-ray findings reported here were merely based on the radiologist report. Compared to pediatric pulmonologist readings, the use of radiologic criteria for diagnosing pneumonia could be suboptimal. Bronchiectasis was found in 8 patients: of whom 2 had no prior lung disease, 2 had bronchiolitis obliterans with organizing pneumonia (BOOP), 3 had asthma and 1 had unknown past-medical history (Table 2). It is, therefore, possible that a prior lung disease could have contributed to the development of bronchiectasis. In a 10-year follow up study, Simila et al. found 12 of 27 patients with type 7 adenovirus infections developed bronchiectasis [29].

Since only 14% of children had documented long-term follow-up, the observed incidence of chronic lung changes is very likely underestimated. These findings, however, highlight the importance of a close clinical and radiologic follow-up of children with adenovirus LRTI. Further prospective studies are required to investigate which risk factors are associated with these long-term changes.

The findings here need to be interpreted with some caution. First, adenovirus testing was performed in a hospital setting, which might overestimate the serious nature of adenovirus infections. Second, like most retrospective epidemiological studies, data are often incomplete and analyses can be subject to bias.

## Conclusions

The epidemiologic, clinical, radiologic features and outcome of adenovirus LRTI in Aboriginal and Non-Aboriginal children in Manitoba, Canada are discussed. Significant respiratory morbidities were associated with adenovirus infections, especially in young infants. The infection was more frequent in Aboriginal children. Significant chronic lung changes may occur following adenovirus infection, emphasizing the need for long-term follow-up.

## Competing interests

The authors declare that they have no competing interests.

## Authors' contributions

SA, PVC and RCA participated in study design and data acquisition. TZ performed statistical analysis. SF participated in data acquisition. AKS participated in manuscript writing. ARA performed study design, data acquisition, data analysis and manuscript writing. All authors read and approved the manuscript.

## Note

Communicated by Alharbi et al

## Pre-publication history

The pre-publication history for this paper can be accessed here:

http://www.biomedcentral.com/1471-2334/12/55/prepub
